# Automated segmentation of microtomography imaging of Egyptian mummies

**DOI:** 10.1371/journal.pone.0260707

**Published:** 2021-12-15

**Authors:** Marc Tanti, Camille Berruyer, Paul Tafforeau, Adrian Muscat, Reuben Farrugia, Kenneth Scerri, Gianluca Valentino, V. Armando Solé, Johann A. Briffa

**Affiliations:** 1 Dept. of Comm. & Computer Engineering, University of Malta, Msida, Malta; 2 European Synchrotron Radiation Facility, Grenoble, France; 3 Dept. of Systems & Control Engineering, University of Malta, Msida, Malta; University of Otago, NEW ZEALAND

## Abstract

Propagation Phase Contrast Synchrotron Microtomography (PPC-SRμCT) is the gold standard for non-invasive and non-destructive access to internal structures of archaeological remains. In this analysis, the virtual specimen needs to be segmented to separate different parts or materials, a process that normally requires considerable human effort. In the Automated SEgmentation of Microtomography Imaging (ASEMI) project, we developed a tool to automatically segment these volumetric images, using manually segmented samples to tune and train a machine learning model. For a set of four specimens of ancient Egyptian animal mummies we achieve an overall accuracy of 94–98% when compared with manually segmented slices, approaching the results of off-the-shelf commercial software using deep learning (97–99%) at much lower complexity. A qualitative analysis of the segmented output shows that our results are close in terms of usability to those from deep learning, justifying the use of these techniques.

## Introduction

For at least a decade, archaeologists have been interested in applying new technologies to their research to better understand our past. State-of-the-art Propagation Phase Contrast Synchrotron Microtomography (PPC-SRμCT) is becoming a golden standard for a non-invasive and non-destructive access to internal structures of archaeological remains. This technique has been recently applied to archaeozoological studies of mummified animal remains from the Ptolemaic and Roman periods of ancient Egypt (around 3^rd^ century BC to 4^th^ century AD). Researchers performed virtual autopsies and virtual unwrapping, uncovering information about animal life and death in past civilisations, as well as revealing the processes used to make these mummies [1, 2]. However, this can be a long process. After microtomographic data processing and reconstruction, the virtual specimen has to be segmented to separate the different parts or different materials of the sample. For biological samples, such as animal mummies, segmentation is usually done semi-manually, and can require weeks of human effort, even for small volumes. Effective automatic segmentation, most likely based on Artificial Intelligence (AI), could drastically reduce this effort, with the expected computation time reduced to a matter of hours, depending on the size and complexity of the volumetric image. This is particularly relevant as the number and sizes of volumes increases. For example, the European Synchrotron Radiation Facility (ESRF) is currently generating huge amounts of data of human organs (up to 2 TiB for a single scan); only AI approaches can handle segmentation at this scale.

In computer vision, image segmentation is an important step in providing the input to higher level tasks such as image understanding, by dividing an image into meaningful regions. Automatic segmentation algorithms have been developed for CT images in the domains of medical imaging, geology and materials analysis, with recent works considering the use of both supervised and unsupervised techniques in 3D tomography. Classical methods, using unsupervised techniques, are generally based on clustering [3] or deformable models [4], with the latter being more resilient to inter-slice deformations and intrinsically accommodate 3D models. In an example from materials analysis [5], cement microtomography images were segmented using self-organizing maps based on neighbourhood features, outperforming K-means clustering and the classical edge operators. Geometric segmentation in 2D and 3D was used in [6] to exploit differences in the geometry of crack formation as opposed to naturally occurring voids. In medical imaging, pelvic bone segmentation from 2D CT slices was reported in [7], where segmentation was achieved following a series of steps which included enhancement, median filtering, smoothing, and contour detection. A review of techniques used for segmentation of the pelvic cavity is available in [8]. Clustering was also used in [9] for segmentation of CT images containing abdominal aortic aneurysms, using spatial fuzzy C-means.

More recently, fully convolutional networks (FCN) were trained to process 3D images in order to produce automatic volumetric semantic segmentation of medical images [10, 11]. The area of biomedical image segmentation is of particular interest to this project because in both cases there is limited availability of ground truth data. This poses a significant challenge for the application of deep learning techniques, which generally require a considerable amount of training data. In an attempt to counteract this problem, data augmentation with elastic deformations was used in [11] to augment the training data set. Unfortunately, there is a significant difference between conventional CT scans and the volumetric images captured by microtomography, both in terms of resolution and contrast. This means that algorithms developed for conventional CT scans are unlikely to work in our application without significant adaptation. Finally, while research in semantic segmentation of 2D images and videos is mature [12], methods applicable to 3D imagery are limited [13, 14]. These methods are not directly applicable to our application, as they consider only 3D point cloud data rather than volumetric scans (voxels). Furthermore, these algorithms were trained and optimized to segment indoor scenes or street scenes, rather than CT images.

Automatic segmentation of volumetric images is most well-developed in medical imaging, such as the CHAOS Challenge [15] task for segmenting the liver from a human CT scan, where many of the top-ranking methods are variations on the deep learning neural network U-Net [11]. By contrast, in archaeology, the problem is less well-studied, and the techniques available are less sophisticated. For example, Hati et al. [16] segmented an entire human mummy into jewels, body, bandages, and the wooden support frame, using a combination of voxel intensity and the manual selection of geodesic shapes. This method has the disadvantage that it is only suitable for segmenting elements that have a high contrast or well-defined edges. For example, the method in [16] did not distinguish between soft tissue, bones, and teeth within the mummy. Low resolution volumetric scans of mummies were considered in [17, 18], with segmentation performed one slice at a time using classical machine learning techniques. In both works, ridged artifacts and loss of detail were evident in the results obtained.

The automatic segmentation in commercially available software for volumetric editing, such as Dragonfly [19], requires a human expert to manually segment a small subset of slices from the tomography image with all the parts that need to be identified. A machine learning model is then trained from the manually segmented examples and used to segment the rest of the volume.

In the Automated SEgmentation of Microtomography Imaging (ASEMI) project, we developed a fully automatic segmenter using the same methodology, based on classical machine learning, which we present here. Our approach has a number of advantages over the state of the art, both in scientific publications and commercial solutions. The main contributions of our work are the following:

- Our segmenter is not limited by the computer’s main memory, allowing it to segment arbitrarily large volumes (e.g. the largest scan presented here requires 133 GiB for the volume itself, and a further 66 GiB for the labels, while the feature vectors would require several TiB).
- The parameters of our segmenter are automatically optimised for the given volume, minimising user input.
- Our segmenter works directly with three dimensional features, rather than reducing the problem to an independent segmentation of two-dimensional slices, without increasing computational complexity from quadratic to cubic. This allows our system to scale well, particularly for larger volumes.
- Our system can use interpretable machine learning models, making it possible to inspect the reasons behind segmentation errors.

Experimental results based on four specimens of animal mummies have an overall accuracy of 94–98% for our proposed system, when compared with manually segmented slices. This approaches the results of off-the-shelf commercial software using deep learning (97–99%) at much lower complexity. A qualitative analysis of the outputs shows that our results are close in terms of usability to those from deep learning. Some postprocessing is necessary to clean up segmentation boundaries, for both our system and the deep learning approach.

Advances in automatic segmentation can be directly applied to a wide variety of other application domains where microtomography is an important tool. In industrial applications automatic segmentation will be useful in non-destructive metrology and detection of voids, cracks and defects. In biomedical research, it would be useful in small animal and tumour imaging, amongst others. Other applications include nanotechnology, geology, electronics and food science. The main objective of this work is to develop and use artificial intelligence techniques to automatically segment volumetric microtomography images of Egyptian mummies, and label each voxel as textiles, organic tissues or bones. The main advantages of our proposed system are that it can achieve high accuracy at lower complexity, allowing it to scale well for very large volumes.

All software and datasets used to generate the results in this work are available for free download. The input and output datasets are available under a Creative Commons Attribution-NonCommercial-ShareAlike 4.0 International License at the ESRF heritage database for palaeontology, evolutionary biology and archaeology [20]. The source code has been released under the GNU General Public Licence (GPL) version 3 (or later) and can be found, together with its documentation, on GitHub [21].

## Algorithm design

### Overview

The ASEMI segmenter uses an algorithm that determines, for every voxel in a volumetric image, a label that corresponds to the material or part that the voxel belongs to. For any given volumetric image there may be a number of labels *N* ≥ 2, where one of the labels represents those parts or materials that are not further distinguished (this label can be interpreted as ‘background’ or ‘anything else’). The algorithm works by first obtaining a descriptive feature vector for every voxel, and then using a classifier to determine a label based on the feature vector.

### The voxel neighbourhood

**Fig 1:**
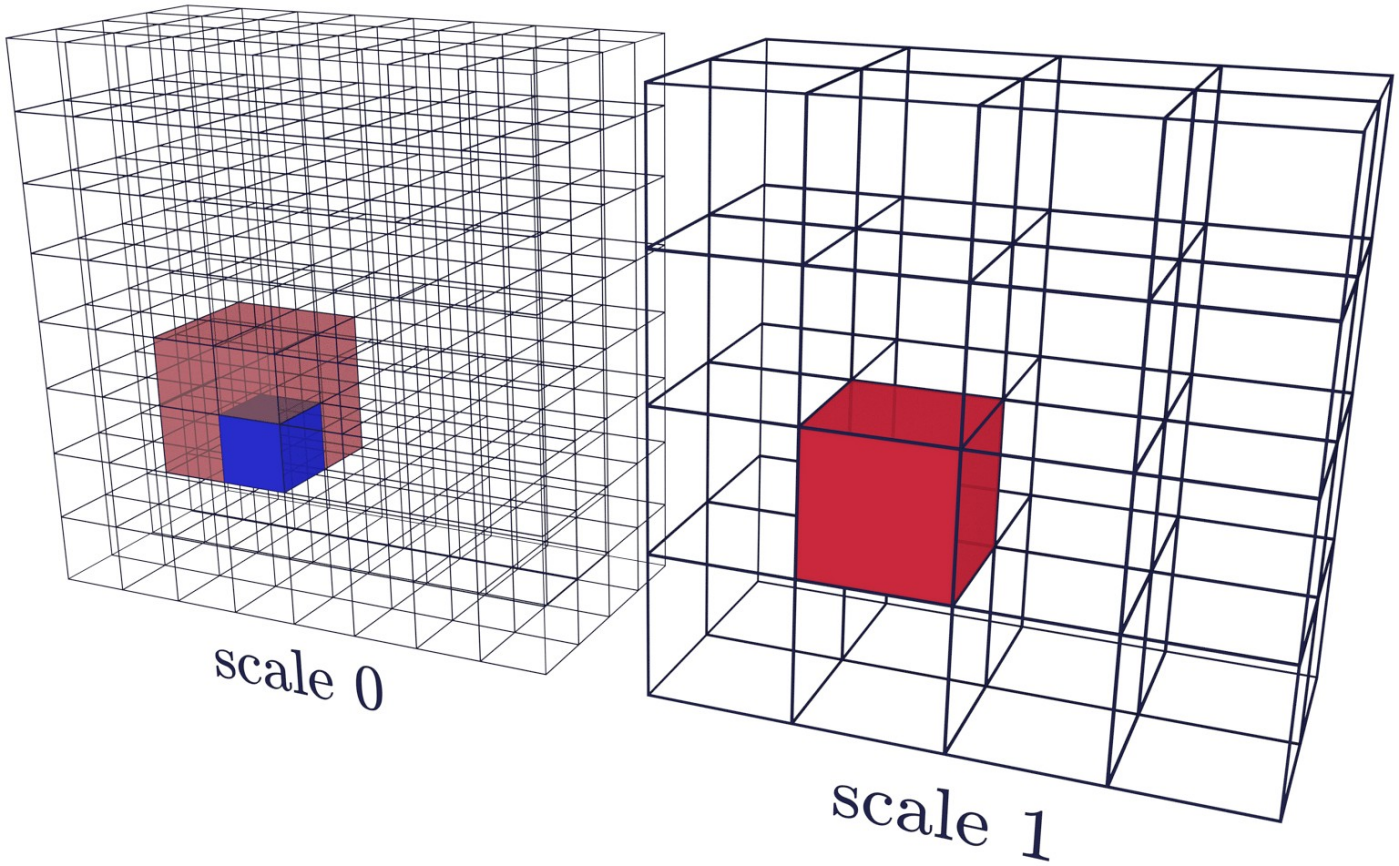
Voxels at different scales. An illustration of a voxel at scale 0 (blue) and its corresponding voxel at scale 1 (red). A voxel at scale 1 (red) corresponds to every voxel in a 2 × 2 × 2 region at scale 0 (light red).

The feature vector for a given voxel depends on the voxel itself and also on the voxel’s context, which is necessary to determine the texture that a voxel forms part of. Formally, the feature vector is a function of the voxel neighbourhood at a given scale, which is defined as follows. The neighbourhood of radius *r* is the cube of voxels of side 2*r* + 1 centered around the voxel of interest. The scale *s* is the number of times the entire volume is resized by half, so that a neighbourhood can be from the original volume (*s* = 0) or from a smaller version of the volume at the corresponding location of the central voxel. These relationships are illustrated in Fig 1. The scaled volumes are obtained by first applying a 3D Gaussian blur to the entire volume and then decimating this by a factor of 2^*s*^ in each dimension. This creates versions of the volume with less detail, allowing the algorithm to operate on larger-scale textures.

### Feature vectors

For any given voxel neighbourhood radius and scale, a number of different features may be obtained. We usually use a concatenation of several feature vectors, each obtained at a different neighbourhood radius and scale, as an input to the classifier.

The simplest feature is the intensity value of the voxel being classified, which corresponds to the density of the material in that location. Clearly, this feature is independent of the neighbourhood radius, and does not carry any information about the material texture. While it is possible to consider the intensity at different scale, we usually use this feature only at scale zero. Therefore, no parameters are necessary to uniquely define this feature.

Next up in complexity is the histogram of intensities within the given neighbourhood. This feature vector consists of an array of *k* frequencies, one for each linearly-spaced bin of voxel intensities. This contains information about the distribution of material density in the given neighbourhood. Computation of this feature vector is defined by three parameters: the neighbourhood radius *r*, scale *s*, and number of bins *k*.

**Fig 2:**
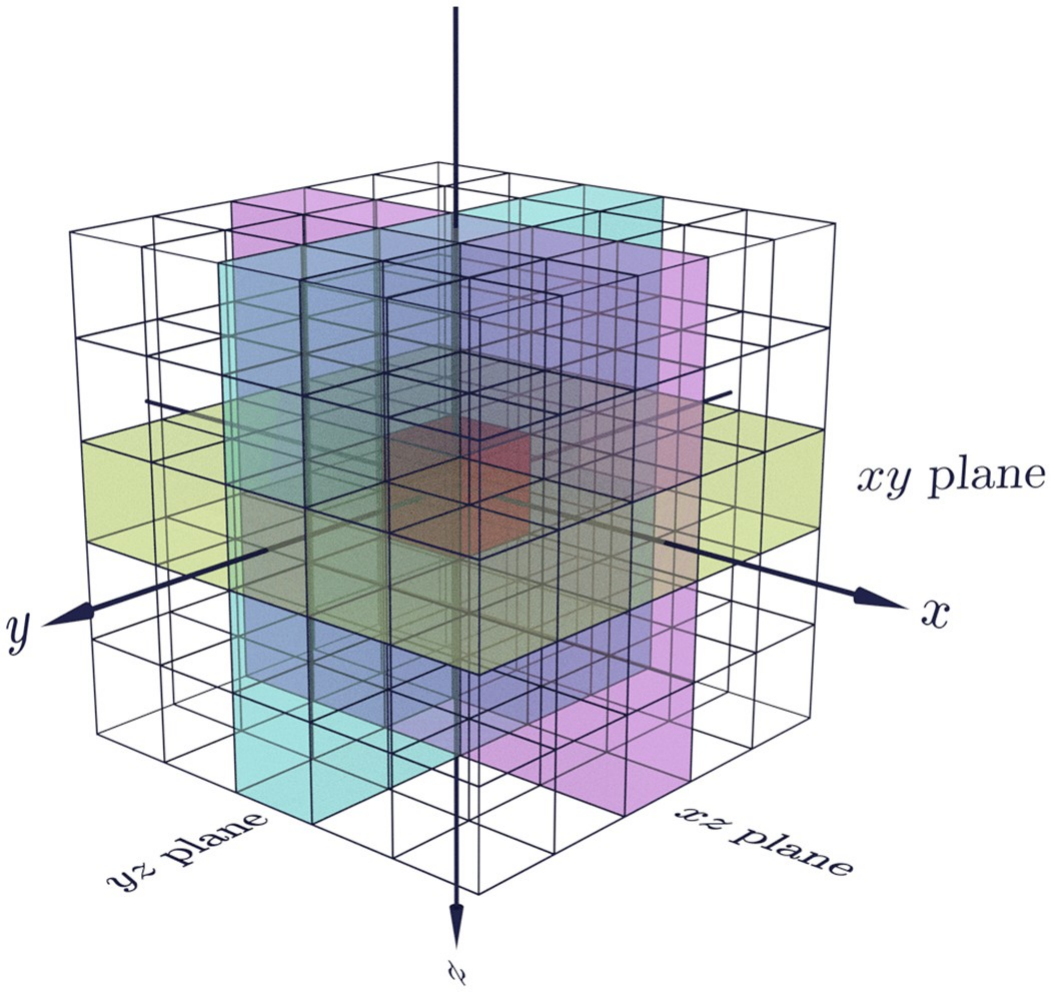
Three orthogonal planes within a voxel neighbourhood. An illustration of the three orthogonal planes in the neighbourhood of radius *r* = 2 for the red voxel in the center. The *xy*, *yz*, and *xz* planes are shown in magenta, cyan, and yellow respectively.

The distribution of material density is one aspect of the texture that a voxel forms part of. Another aspect is the structure of the texture. This is captured well by the Local Binary Pattern (LBP) [22], which operates on a plane, and is insensitive to density variation but sensitive to the relative location of voxel values. In order to capture information in three dimensions, we combine the features from three orthogonal planes centered on the voxel under inspection [23, 24], as illustrated in Fig 2 for a neighbourhood of radius *r* = 2. First, an LBP code is obtained for every voxel, based on its immediate 3 × 3 neighbourhood in the plane considered (i.e. the 8 neighbours at radius 1). Uniform and rotation invariant LBP codes [25] are used, as preliminary experiments showed that these improve performance while reducing the feature vector size. Next, we obtain a histogram of the codes corresponding to voxels in a 2D neighbourhood of radius *r* in the plane considered, and use this as our feature vector. Computation of this feature vector is defined by three parameters: the scale *s* and histogram neighbourhood radius *r*. The number of histogram bins is fixed to 10, as that is the number of different codes possible.

Other features exist for encoding textural information, such as SIFT [26] and SURF [27], which have found extensive use in computer vision. In preliminary experiments we found that these features were computationally much more expensive and did not improve performance. Therefore, we did not consider them further for this application.

The final feature vector chosen is the concatenation of: a) the value of the voxel being classified, b) a short range histogram of voxel values at scale zero, c) a long range histogram of voxel values (at a scale to be determined), and d) a three orthogonal planes LBP (at a scale to be determined). Preliminary experiments showed that the combination of histogram and LBP features was better than either one alone. We also experimented with two-dimensional histograms across the three orthogonal planes, as well as the mean value of the voxel neighbourhood, but the performance increase was minimal and did not justify the added complexity. Using both short and long range features (both histogram and LBP) resulted in a small performance increase over using only long range features. Rather than use long and short range features for both histogram and LBP, we opted to limit this to the histogram features which are faster to compute.

### Classifier

The extracted features are used as input to a classifier, which is trained to create a mapping between the feature vectors and the corresponding label. Two classifiers are used in this work: the random forest classifier [28], as implemented in scikit-learn [29], and the feedforward neural network [30] implemented in Tensorflow [31]. The random forest classifier has the advantage of being an interpretable model while the neural network generalises well and has a faster GPU-based implementation. We also experimented with support vector machines [32], but these were not performing as well as the other methods so we did not consider them further.

The random forest classifier was trained with a node splitting criterion based on the Gini impurity, no limit on the number of leaf nodes, a maximum tree depth of 16, and the number of features per tree equal to the square root of the total number of features. The only remaining hyperparameter, the number of trees, was left as a free variable to be determined in the hyperparameter optimisation process, within the range specified in Table 1.

**Table 1:** Search space for free variables in feature selection and model hyperparameters.

**a**: Feature selection.		
*Feature*	*Variable*	*Search Space*
Histogram 1	Radius	1-8
	Bins	{8, 16, 32}
Histogram 2	Scale	0-2
	Radius	1-32
	Bins	{8, 16, 32}
LBP	Scale	0-2
	Radius	1-32
**b**: Model hyperparameters.		
*Classifier*	*Hyperparameter*	*Search Space*
Random forest	Number of trees	{16, 32, 64}
Neural network	Layer 1 size	{32, 64, 128, 256}
	Layer 2 size	{32, 64, 128, 256}
	Dropout rate	0.0-0.5
	Initialisation stddev.	0.0001-1.0
	Minibatch size	{4, 8, 16, 32, 64}

Similarly, the neural network structure included two hidden layers with a leaky ReLU activation function, and was trained with the Adam optimiser, early stopping criterion, dropout, and weight initialisation using normally distributed random numbers with zero mean and bias initialisation of zero. The remaining hyperparameters, left as free variables to be determined in the hyperparameter optimisation process, are listed in Table 1, together with the range explored.

While other more complex hyperparameter optimisation approaches have been used in the literature, in this work we opted for a simple approach. Our optimisation process consists of a two-stage random search in the combined search space of the feature selection variables and model hyperparameters. The first stage randomly samples the entire search space, uniformly across all variables; this explores the search space more efficiently than a grid search at the same resolution. The second stage is a local search, starting with the best result from the first stage. The algorithm randomly changes a single variable, in an iterative process that is similar to a stochastic hill climbing optimiser. Details are outlined in Algorithm 1.

**Algorithm 1**: **Two-stage search algorithm**.

**Input**: A training set and a validation set.

**Data**: A number of global iterations *I*_*g*_ = 1000, a number of local iterations *I*_*l*_ = 10000, a number of training set voxel samples *S*_*t*_ = 16000, a number of validation set voxel samples *S*_*v*_ = 16000, a number of seconds as a timeout limit *t* = 5.

**Output**: An optimised set of variables

sampledTrainSet ← a sample of *S*_*t*_ items from each label in the training set

sampledValSet ← a sample of *S*_*v*_ items from each label in the validation set

knownVarSets ← ⌀

bestVarSet ← null

bestAccuracy ← 0

**begin** global phase

 **for** *I*_*g*_ *times* **do**

  **repeat**

   varSet ← a random set of variables

   **if** *more than t seconds have passed* **then** exit global phase

  **until** *varSet* ∉ *known VarSets*

  add varSet to knownVarSet

  segmenterModel ← new segmenter model using varSet

  train segmenterModel using sampledTrainSet

  modelAccuracy ← evaluate segmenterModel accuracy using sampledValSet

  **if** *modelAccuracy* > *bestAccuracy* **then**

   bestVarSet ← varSet

   bestAccuracy ← modelAccuracy

  **end**

 **end**

**end**

**begin** local phase

 **for** *I*_*l*_ *times* **do**

  **repeat**

   varSet ← bestVarSet

   replace a single variable in varSet with a random value

   **if** *more than t seconds have passed* **then** exit local phase

  **until** *varSet* ∉ *knownVarSet*

  add varSet to knownVarSet

  segmenterModel ← new segmenter model using varSet

  train segmenterModel using sampledTrainSet

  modelAccuracy ← evaluate segmenterModel accuracy using sampledValSet

  **if** *modelAccuracy* > *bestAccuracy* **then**

   bestVarSet ← varSet

   bestAccuracy ← modelAccuracy

  **end**

 **end**

**end**

**Result**: bestVarSet

## Implementation efficiency and scalability

### Overview

For the application considered, with microtomography images of 10^9^ to 10^10^ voxels, the scalability of the algorithms is a critical concern. There are two aspects to this: memory requirement and computational complexity. At this scale, the images are at the limit of what will fit in current high-RAM machines, while the set of feature vectors for the entire volume will easily exceed available memory. Next-generation scans, such as those recently started at the ESRF, generate images that are already larger than available memory. It is therefore necessary for the algorithm implementation to divide the volume into subvolumes and operate on these separately. In a naive implementation, the computational complexity scales linearly with the number of voxels, as the extraction of the feature vector can be seen as independent for each voxel. However, neighbouring voxels have overlapping neighbourhoods, so that some computations can be shared between them. An optimised implementation can leverage this to reduce the overall complexity.

### Optimised histogram computation

The main contributor to the computational complexity of feature extracation is the computation of histograms of voxel neighbourhoods, used for both the histogram feature vector and the LBP feature vector. When the histogram for each neighbourhood is independently computed, the complexity is cubic with respect to the neighbourhood radius *r*. If we incrementally compute histograms for the neighbourhoods of adjacent voxels, however, we can reduce the overall complexity to quasi-quadratic.

**Fig 3:**
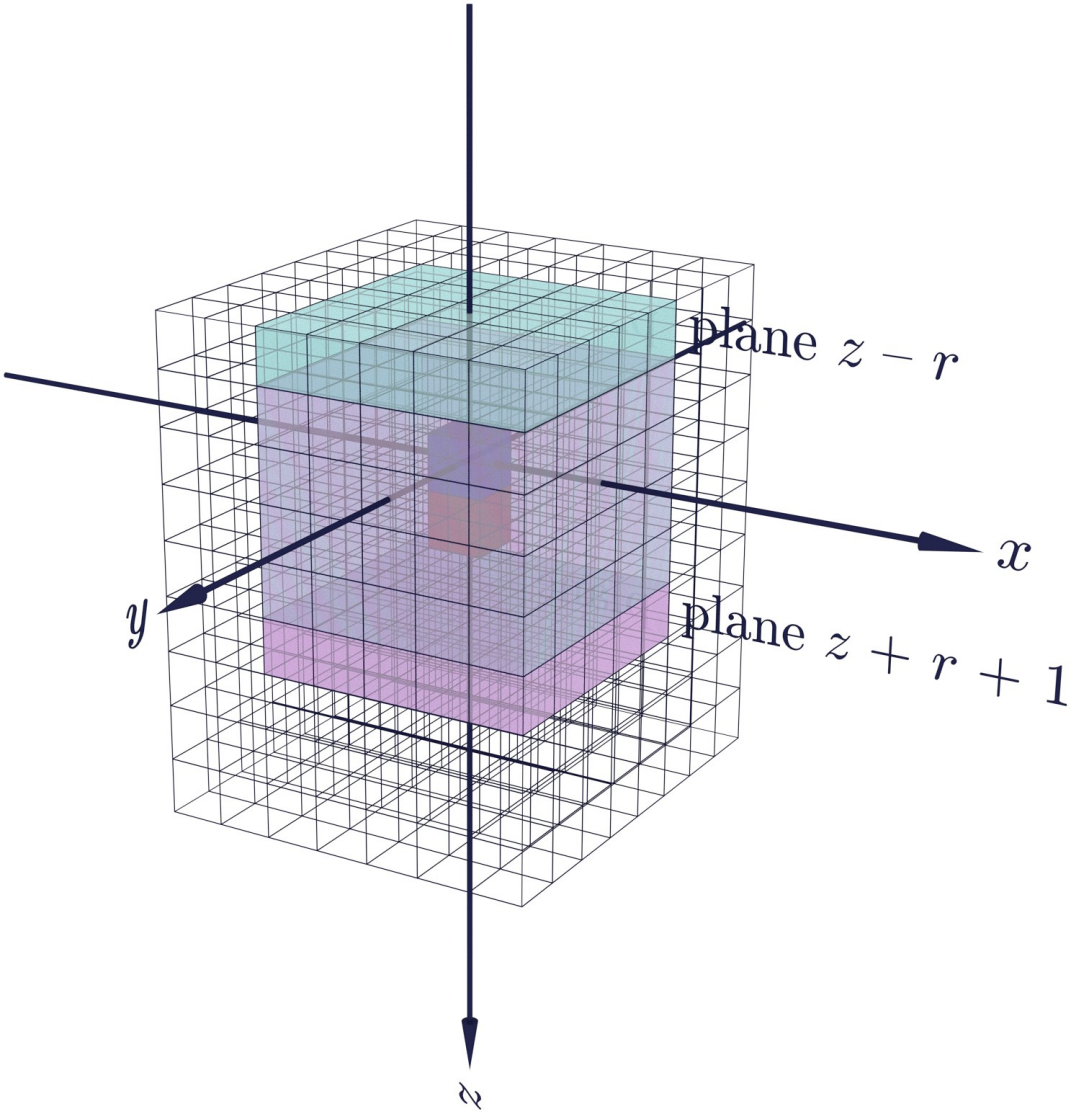
Neighbourhood overlap for adjacent voxels. An illustration of the overlap between the neighbourhoods of radius *r* = 2 for the blue voxel *v*_*x*,*y*,*z*_ (shown in cyan), and the adjacent red voxel *v*_*x*,*y*,*z*+1_ (shown in magenta). The plane *z* − *r* exists only in the first neighbourhood, while the plane *z* + *r* + 1 exists only in the second neighbourhood.

Consider the neighbourhood of radius *r* of a voxel *v*_*x*,*y*,*z*_ at index *x*, *y*, *z* in the volume. Computing the histogram for this single neighbourhood requires consideration of (2*r* + 1)^3^ voxel intensities, for a complexity *O*(*r*^3^). Now consider the neighbourhood of adjacent voxel *v*_*x*,*y*,*z*+1_. This overlaps with the neighbourhood of *v*_*x*,*y*,*z*_, except for the planes *z* − *r* and *z* + *r* + 1, as illustrated in Fig 3. Therefore, we can compute the neighbourhood histogram for *v*_*x*,*y*,*z*+1_ as an incremental change on that of *v*_*x*,*y*,*z*_. Starting with the histogram for *v*_*x*,*y*,*z*_, we subtract the frequencies for the plane *z* − *r* and add the frequencies for the plane *z* + *r* + 1. Each of these two operations requires consideration of (2*r* + 1)^2^ voxel intensities, for an overall complexity *O*(*r*^2^) for this incremental histogram computation.

For any given subvolume, the first neighbourhood histogram needs to be computed independently, while all other histograms can be determined incrementally. Assuming the subvolume size is much larger than *r*, the effect on complexity of the first histogram becomes insignificant, for an overall complexity approaching *O*(*r*^2^).

A considerable improvement in efficiency is also obtained by computing histograms on a GPU, using NVIDIA’s CUDA framework [33]. For incremental histogram computation it is advantageous to consider operations on independent slices. We can compute the histograms for each voxel neighbourhood in parallel, because they are independent. This allow us to take advantage of the massively parallel GPU architecture. Furthermore, observe that even in this case, the neighbourhoods for adjacent voxels overlap significantly. We take advantage of this overlap by loading the neighbourhood into on-chip ‘shared’ memory, which is accessible at cache-level speeds by the computational units of the multiprocessor. This significantly reduces the number of memory reads required, for a significant speedup in this I/O-bound problem.

We also take advantage of a number of GPU architecture features to ensure an efficient implementation. These include the parallel reading of tile neighbourhood data into shared memory, organised so that access by warp threads is on different memory banks. Incrementally computed histograms are also kept in shared memory, writing only the final 3D histogram results to main memory.

Note that these improvements are also of benefit when computing LBP features. Specifically, for each of the three orthogonal planes, once the LBP codes corresponding to each voxel are computed, a histogram of these codes is obtained for the 2D voxel neighbourhood of radius *r*. The computation of the LBP codes takes constant time, as for each voxel it requires a comparison with the eight immediate neighbours. Computing the histogram of the codes requires consideration of (2*r* + 1)^2^ voxel intensities, for a complexity *O*(*r*^2^). The histogram computation therefore dominates the complexity of the LBP feature extraction.

Comparative timings were obtained during development to validate these optimisation steps. Table 2 gives the time taken for the feature extraction process on a small 180 × 150 × 200 volume using an Intel Core i7-8700K CPU at 3.7 GHz and an NVIDIA GeForce GTX 1080 Ti. In all cases, histograms are computed using the incremental algorithm.

**Table 2:** Validating speedups on a small volume. Volume size 180 × 150 × 200; CPU: Intel Core i7-8700K at 3.7 GHz; GPU: NVIDIA GeForce GTX 1080 Ti.

	CPU (s)	GPU (s)
Histogram (*r* = 32, *s* = 0, *k* = 32)	17.8	1.1
LBP (3-plane, *r* = 32, *s* = 0)	24.4	1.3

### Complexity analysis and comparison with U-Net

The overall complexity of the ASEMI segmenter can be determined by considering both the feature extraction and the classifier. The complexity for computing the features depends directly on the neighbourhood radius *r*, as already shown. When incremental histogram computation is used, the complexity for both the 3D histogram and the three-orthogonal-plane LBP features have a complexity *O*(*r*^2^). The complexity of the classifier depends on the number of features computed, as well as on the statistical properties of the data (i.e. how easily separable the data is). The number of features is equal to the number of bins *k* for the histogram features, and has a fixed value of 30 for the three-orthogonal-plane LBP. Due to the dependency on the statistical properties of the data, we determine the complexity for the classifiers empirically, for the cases considered in the Results section. Typical models based on the ASEMI segmenter (random forest and three layer neural network) require around 20-60 k operations per voxel, including the feature computation.

In the U-Net architecture, the size of the context over which the classification decision is computed is taken as equal to the effective receptive field. Since the effective receptive field depends on the complete architecture of the network we compare the complexity empirically for typical architectures from the literature. The 3D U-Net [34] is characterised with 19.07 M parameters and a receptive field of approximately 88 voxels in each direction, equivalent to *r* ≈ 43. Considering only multiplication operations, we determined the number of computations per output voxel to be around 5.4 M operations per voxel. For the 2D U-Net [11], around 640 K operations per voxel are required when *r* ≈ 30.

It is clear from this comparison that the ASEMI segmenter has a significant complexity advantage over deep learning methods. In both cases, much of the computation can be performed on a GPU accelerator. Furthermore, the feature vector sizes of a typical deep neural network are in the order of hundreds whereas our feature vector sizes are in the order of tens, giving our algorithm a potential advantage in GPU memory requirements. These results justify the use of classical methods over deep learning methods for 3D segmentation.

## Experimental setup

### Overview

**Fig 4:**
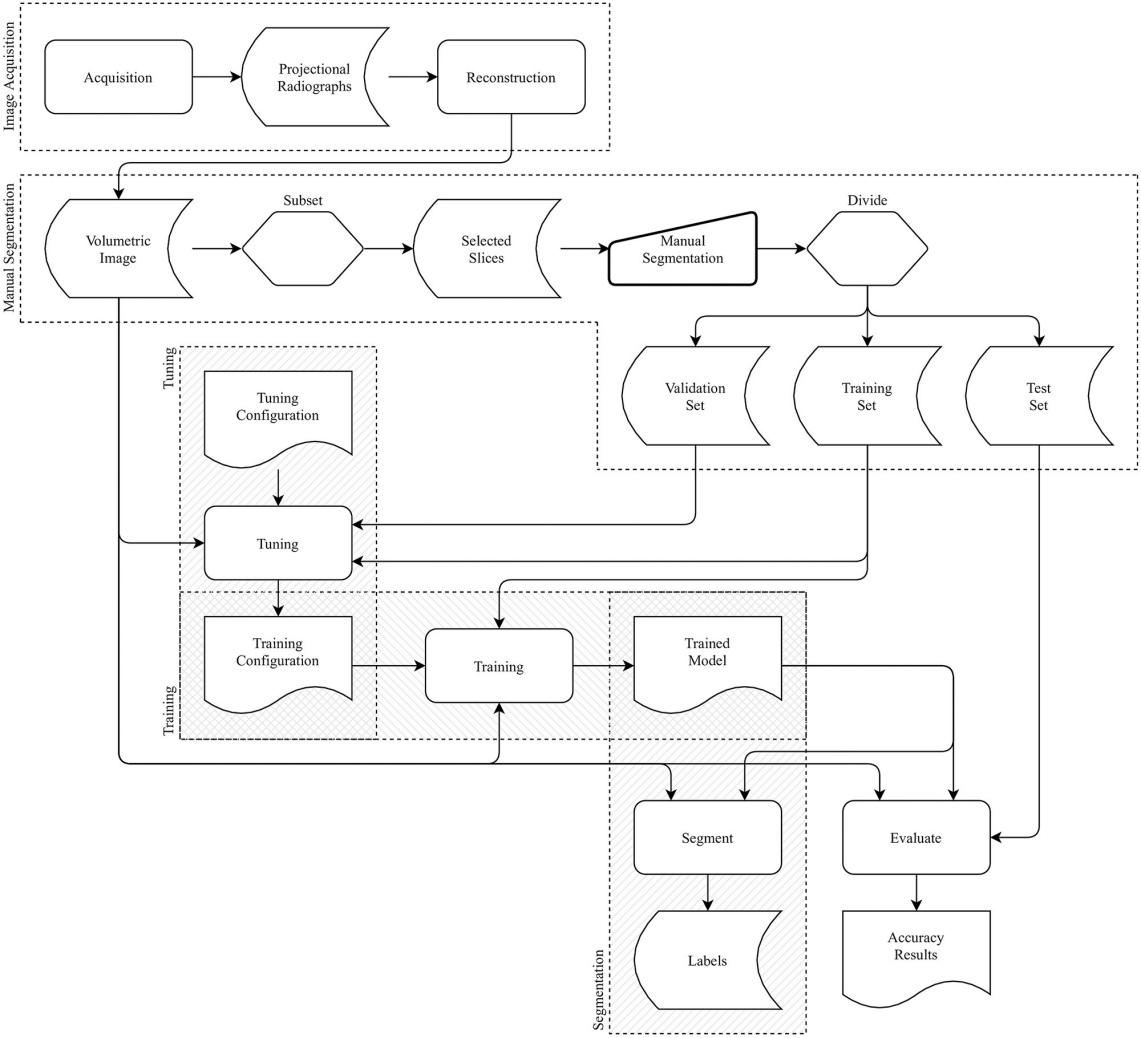
Block diagram of the overall process, from scanning to automatic labelling.

Experimental results, based on four specimens, compare the performance of the ASEMI segmenter with U-Net. The overall process, from scanning each specimen to its automatic labelling, is shown in Fig 4. Following image acquisition and reconstruction, a selection of slices from each volumetric image are manually segmented and divided into three independent sets, for training, validation, and testing of the machine learning models obtained. For the ASEMI segmenter, a feature selection and model hyperparameter optimisation process uses a subset of the training and validation sets to determine the best set of features and classifier parameters, which are then used to train the machine learning model. For U-Net, these two steps are combined. In both cases, the trained model is then used to segment the complete volume. For final presentation, the automatic segmentations were cleaned and rendered using VGStudioMax, which is excellent for visualisation and rendering. The trained model is also evaluated by determining the classification accuracy in comparison with the previously withheld manually segmented test set. Details of the specimens used and of each of these steps are given in the rest of this section.

### Specimens

**Fig 5:**
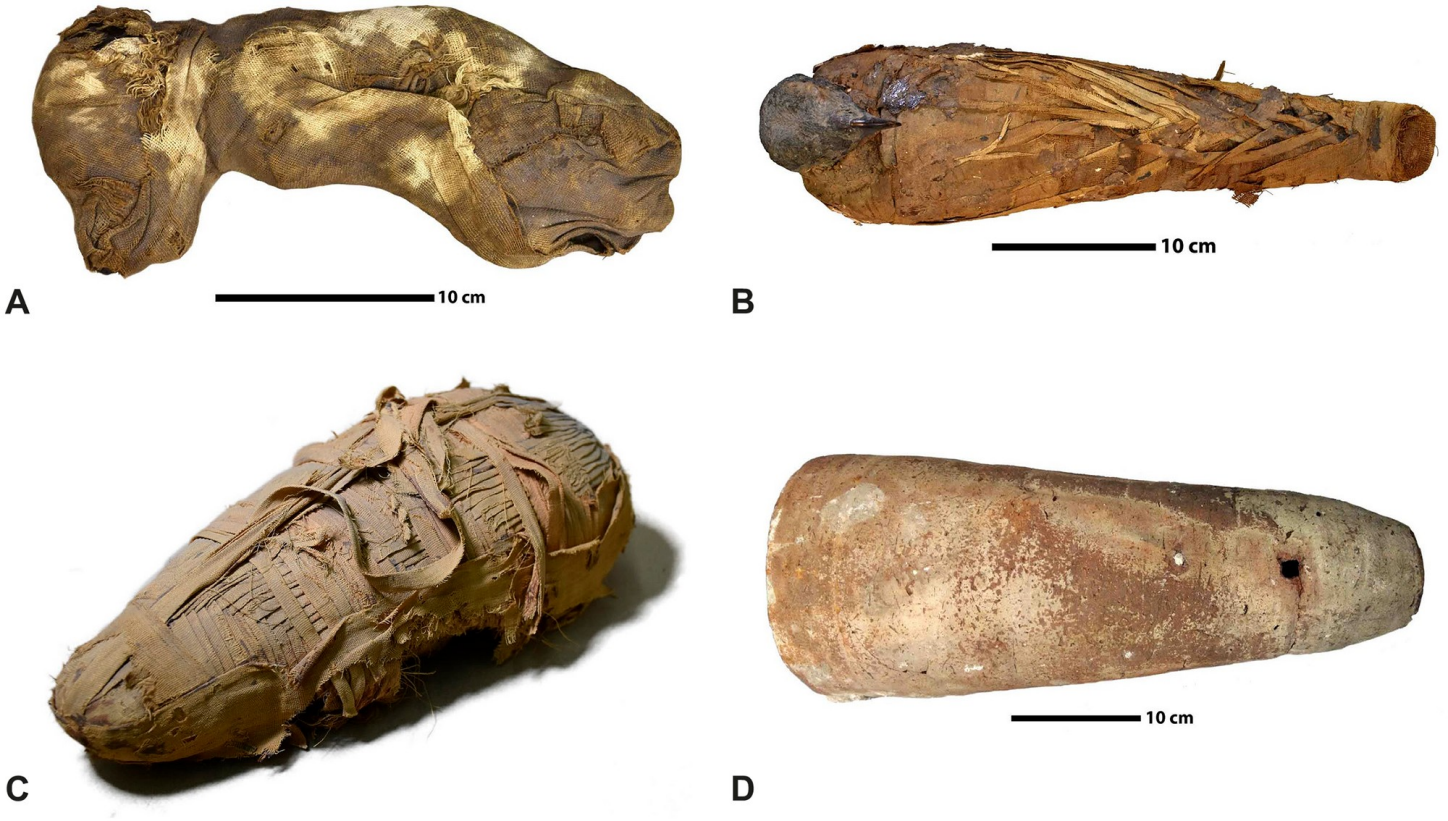
The four specimens used in our experiments, identified by their museum accession numbers. A: MHNGr.ET.1023 (dog), B: MHNGr.ET.1017 (raptor), C: MHNGr.ET.1456 (ibis), D: MG.2038 (ibis in a jar).

The four specimens used in this study are mummified animals from ancient Egypt. They are curated by the Museum d’Histoire Naturelle de Grenoble (Fig 5A–5C) and the Musée de Grenoble (Fig 5D). Like many other animal mummies kept in museums, we know very few things about their precise historical and archaeological origins. They are estimated from Ptolemaic and Roman period (around 3^rd^ century BC to 4^th^ century AD). The first specimen, MHNGr.ET.1023, is a mummy of a puppy. Traces on its fabrics show that it was wrapped with more bandages than its actual state. The second specimen, MHNGr.ET.1456, is the mummy of an ibis still wrapped in elaborate wrappings. Third specimen, MG.2038, is the mummy of an ibis enclosed inside a ceramic jar which was sealed with a type of mortar. The fourth mummy, MHNGr.ET.1017, is a mummy with a raptor bird’s head directly visible and a wrapped body. Unlike the other specimens, the volumetric image doesn’t contain the whole mummy, but focuses only on the stomach area of the bird inside the mummy.

### Image acquisition

The animal mummies used in this work were scanned at the European Synchrotron Radiation Facility (ESRF) in Grenoble, France. The properties of the synchrotron source allows Propagation Phase Contrast tomography, which gives images with higher resolution and better contrast than conventional sources. In our case, three configurations were used, from 24.19 μm to 50.72 μm voxel size. For each scan, the volumetric images were reconstructed using a single distance phase-retrieval algorithm coupled with filtered back projection implemented in the PyHST2 software package [35, 36] and ring artefact corrections were applied. Then, the scanned sections were vertically concatenated and the final files were saved in 16-bit JPEG2000 format, using lossy compression with a target compression rate of 10, four levels of wavelet decomposition, and a tile size that covers the entire image. Detailed scan parameters are summarized in Table 3.

**Table 3:** Scan parameters used to scan the animal mummies at the ESRF.

Voxel size (μm)	50.72	47.8	24.19
Sample	MG.2038 (ibis in a jar) complete mummy, MHNGr.ET.1456 (ibis) complete mummy	MHNGr.ET.1017 (raptor) complete mummy	MHNGr.ET.1023 (dog) complete mummy
Optic	Lafip 2—Hasselblad 100	47 micron ID17	Lafip 2
Date	12 December 2017	18 November 2017	19 November 2017
Average detected energy (keV)	≈ 129	≈ 107	≈ 110
Filters (mm)	Cu 6, Mo 0.25	Al prof 18 × 5, Mo 0.2	Al pro 18 × 5, Mo 0.25
Propagation distance (mm)	4500	4000	3000
Sensor	FReLoN 2k14	FReLoN	PCO Edge 4.2 CLHS
Scintillator	Scintillating fiber	LuAg 2000	LuAg 2000
Projection number	5000	5000	5000
Scan geometry	Half Acquisition 950 pixels offset, vertical scan series with 2.5 mm steps	Half Acquisition 500 pixels offset, vertical scan series with 2 mm steps	Half Acquisition 300 pixels offset, vertical scan series with 2.3 mm steps
Exposure time (s)	0.04	0.03	0.015
Number of scan	180	191	126 + 126
Reconstruction mode	Single distance phase retrieval [36], vertical concatenation, ring artefacts correction, 16 bit conversion in JPEG 2000 format, binning	Single distance phase retrieval [36], vertical concatenation, ring artefacts correction, 16 bit conversion in JPEG 2000 format, binning	Single distance phase retrieval [36], vertical concatenation, ring artefacts correction, 16 bit conversion in JPEG 2000 format, binning

### Manual segmentation

As mentioned earlier, manual segmentation is the most human time-consuming step. The speed and accuracy of the manual segmentation process depends primarily on the complexity of the specimen and the quality of its tomographic data, as well as on the individual performing the segmentation. In our case, a manual selection with active grey level range was used in order to segment a selection of slices in Dragonfly. This step should not be affected by mislabelling or ambiguities except from human error.

The manually segmented slices were split into a training set, a validation set, and a test set using 60%, 20%, and 20% of the slices respectively. The training and validation sets were used to optimise (for ASEMI) and train the machine learning model, while the test set was used only to determine the model accuracy.

### Automatic segmentation with ASEMI

The process for automatic segmentation with ASEMI starts by optimising the feature selection and model hyperparameters for each specimen, using a sample taken from the training and validation sets. A new model is trained using the optimised features and model hyperparameters on the full training set. This model is then evaluated on the full test set, and is also used to segment the whole volume.

The optimised features and model hyperparameters for each classifier and specimen are given in Table 4. Observe that the random forest always opted for the maximum allowed number of trees, and the neural network always opted for a minibatch size just below the maximum allowed. The LBP and Histogram 2 features tended to select mid-range to large neighbourhoods, with both classifiers.

**Table 4:** Optimised features and model hyperparameters.

**a**: Random forest classifier.					
*Feature*	*Variable*	*MHNGr.ET.1023 (dog)*	*MHNGr.ET.1017 (raptor)*	*MHNGr.ET.1456 (ibis)*	*MG.2038 (ibis in a jar)*
Histogram 1	Radius	3	6	5	7
	Bins	32	8	8	32
Histogram 2	Scale	0	0	0	2
	Radius	12	27	21	24
	Bins	16	32	32	16
LBP	Scale	0	1	1	0
	Radius	15	27	20	30
Random forest	Number of trees	64	64	64	64
**b**: Neural network classifier.					
*Feature*	*Variable*	*MHNGr.ET.1023 (dog)*	*MHNGr.ET.1017 (raptor)*	*MHNGr.ET.1456 (ibis)*	*MG.2038 (ibis in a jar)*
Histogram 1	Radius	7	8	7	7
	Bins	16	32	8	32
Histogram 2	Scale	1	2	0	2
	Radius	6	32	27	29
	Bins	8	32	32	16
LBP	Scale	1	1	1	0
	Radius	18	28	27	28
Neural network	Layer 1 size	256	256	64	256
	Layer 2 size	64	64	128	64
	Dropout rate	0.4	0.45	0.25	0.25
	Init. stddev.	0.0001	0.0251	0.1585	0.0016
	Minibatch size	32	32	32	32

### Automatic segmentation with U-Net

The deep learning tool in Dragonfly was used to create a 5-level U-Net model with the required number of output labels. The model was trained on the same training and validation sets used with the ASEMI segmenter, and evaluated on the same test set. For generating the full volume segmentation, however, a separate model was trained with the full set of manually segmented slices, reserving 25% of patches for validation.

Dragonfly does not provide direct tools for hyperparameter optimisation, so we used the hyperparameters chosen by the ESRF annotators, as follows. In all cases, the data was augmented with a flip in each direction and also with a rotation of up to 180°. A patch size of 128 was used, with a stride to input ratio of 0.5, batch size 64, the categorical crossentropy loss function, and Adadelta optimization algorithm, over 100 epochs.

### Volumetric image parameters and timings

For each of the four specimens, Table 5 shows the size of the volumetric image with the number of slices chosen for manual segmentation and the number of labels used. The time taken for manual segmentation and for each step in the automatic segmentation process are also included. Timings for ASEMI were obtained on a system with an Intel Xeon W-3225 CPU at 3.70 GHz, 256 GiB DDR4-2666 RAM, and an NVIDIA GeForce RTX 2080 Ti GPU. Timings for U-Net were obtained on a system with a dual Intel Xeon Platinum 8160 CPU at 2.1 GHz, 1.5 TiB DDR4-2666 RAM, and two NVIDIA Quadro P6000 GPUs. It can be readily seen that the training time for ASEMI is already significantly faster than for U-Net, even though the ASEMI implementation has only been partially optimised, while U-Net is based on the heavily optimised Tensorflow. Unfortunately, the segmentation time is still considerably slower in ASEMI, due to the current architecture of the implementation, which works off disk. We believe it is possible to significantly improve this aspect of the implementation, without losing the advantage of being able to work with volumes much larger than memory, and without any changes to the algorithm performance.

**Table 5:** Volumetric image parameters and time spent on the various stages of segmenting the images.

		*MHNGr.ET.1023 (dog)*	*MHNGr.ET.1017 (raptor)*	*MHNGr.ET.1456 (ibis)*	*MG.2038 (ibis in a jar)*
Volume parameters	Volume size (× 10^9^ voxels)	27.2	71.2	41.5	17.1
	Number of manually segmented slices	18/35	22	21	22
	Number of labels (excluding null)	4	7	8	7
Manual	Segmentation time (h)	4		6	5
U-Net	Training time (h)	15		38	20
	Segmentation time (h)	3		12	5
ASEMI	Optimisation time (h)	29.0	45.5	52.9	33.5
	Training time (h)	6.5	10.2	13.6	6.6
	Segmentation time (h)	67.7	218.7	113.6	53.0
Post-processing	Time to transfer to VGStudioMax (h)	2		4	3
	Time to clean segmented output (h)	2		0	0

## Results

### Accuracy comparison

An initial comparison of the different machine learning models is based on the overall accuracy of the trained model when used to segment parts of the volume not already seen during training. Accuracy is given by the intersection-over-union metric, which we compute for the three models considered, as shown in Table 6. The same test set is used for each model, ensuring comparability of results. It is readily seen that U-Net outperforms ASEMI in almost every label. Interestingly, the few cases where ASEMI performs better include those involving labels with a small representation: ‘teeth’ in MHNGr.ET.1023 (dog) and ‘wood’ in MHNGr.ET.1017 (raptor). In these cases it seems that the neural network classifier with ASEMI features is better able to learn an association with less training data. It would be interesting to see whether this behaviour allows us to achieve reasonable performance with less manually segmented slices. Another instance where ASEMI performs better is ‘bones’ in MG.2038 (ibis in a jar). In this case we know that there are some errors in the manual segmentation; it seems that in such cases the random forest classifier with ASEMI features is able to correct these errors, as we will see in the following analysis.

**Table 6:** Intersection-over-union results for individual labels and overall accuracy for four specimens. U-Net: Dragonfly implementation; RF: ASEMI segmenter with random forest classifier; NN: ASEMI segmenter with neural network classifier.

*Label*	*MHNGr.ET.1023 (dog)*			*MHNGr.ET.1017 (raptor)*			*MHNGr.ET.1456 (ibis)*			*MG.2038 (ibis in a jar)*		
	*U-Net*	*RF*	*NN*	*U-Net*	*RF*	*NN*	*U-Net*	*RF*	*NN*	*U-Net*	*RF*	*NN*
Bones	**92.2%**	76.2%	85.5%				**88.7%**	86.2%	81.9%	93.4%	**96.0%**	90.5%
Teeth	43.2%	14.5%	**46.6%**									
Feathers							**69.3%**	61.6%	64.9%	**67.7%**	21.5%	28.7%
Soft parts	**87.4%**	70.3%	72.9%	**77.4%**	66.1%	66.4%				**92.8%**	58.8%	81.6%
Soft powder							**74.9%**	58.3%	71.3%			
Stomach				**95.9%**	84.8%	80.4%						
Snails							**87.2%**	60.0%	54.2%			
Textiles	**93.1%**	83.6%	85.1%	**96.5%**	90.8%	91.4%	**86.2%**	82.7%	82.3%			
Balm textile				**85.3%**	81.1%	80.5%						
Dense textile				**81.0%**	66.5%	67.9%	**67.0%**	57.5%	62.8%	**96.5%**	78.0%	78.7%
Natron							**60.4%**	36.9%	22.0%			
Ceramics				**78.8%**	67.7%	65.3%						
Terracotta										**99.8%**	98.9%	99.3%
Cement										**94.4%**	74.8%	78.2%
Wood				84.4%	76.8%	**94.8%**						
Insects							**25.4%**	4.6%	4.5%			
Powder										**96.8%**	70.9%	71.8%
Unlabelled	**99.2%**	97.7%	98.7%	**97.7%**	95.0%	89.9%	**99.0%**	97.1%	98.8%	**99.4%**	97.0%	98.5%
Overall	**98.9%**	96.5%	97.7%	**97.2%**	94.2%	94.3%	**97.4%**	94.6%	96.8%	**99.4%**	96.0%	97.2%

### Error analysis

**Fig 6:**
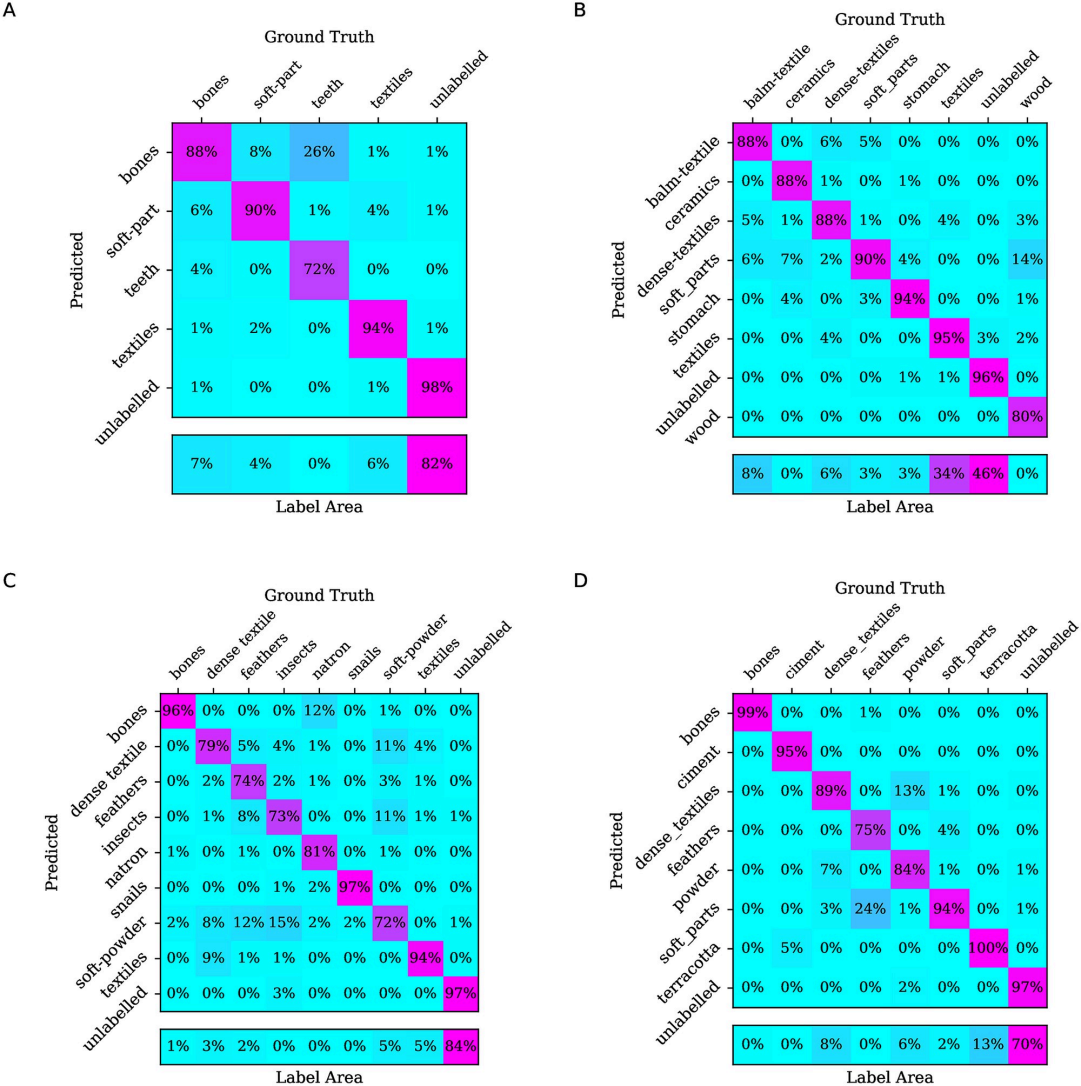
Confusion matrices for random forest predictions. For each specimen’s test set, the confusion matrix is given together with the percentage of the number of voxels in the test set occupied by a given label. A: MHNGr.ET.1023 (dog), B: MHNGr.ET.1017 (raptor), C: MHNGr.ET.1456 (ibis), D: MG.2038 (ibis in a jar).

A quantitative error analysis can be performed by considering the confusion matrices over the test set predictions, as shown in Fig 6 for the random forest classifier. This shows, for every label, what the voxels are classified as. Numbers on the main diagonal indicate correct classification, while non-zero off-diagonal values indicate misclassifications. Fig 6 also includes, for each label, the percentage of the number of voxels in the test set occupied by that label. Some general trends can be observed, particularly that misclassification is more likely for labels that have a low representation, and between labels with similar textures.

**Fig 7:**
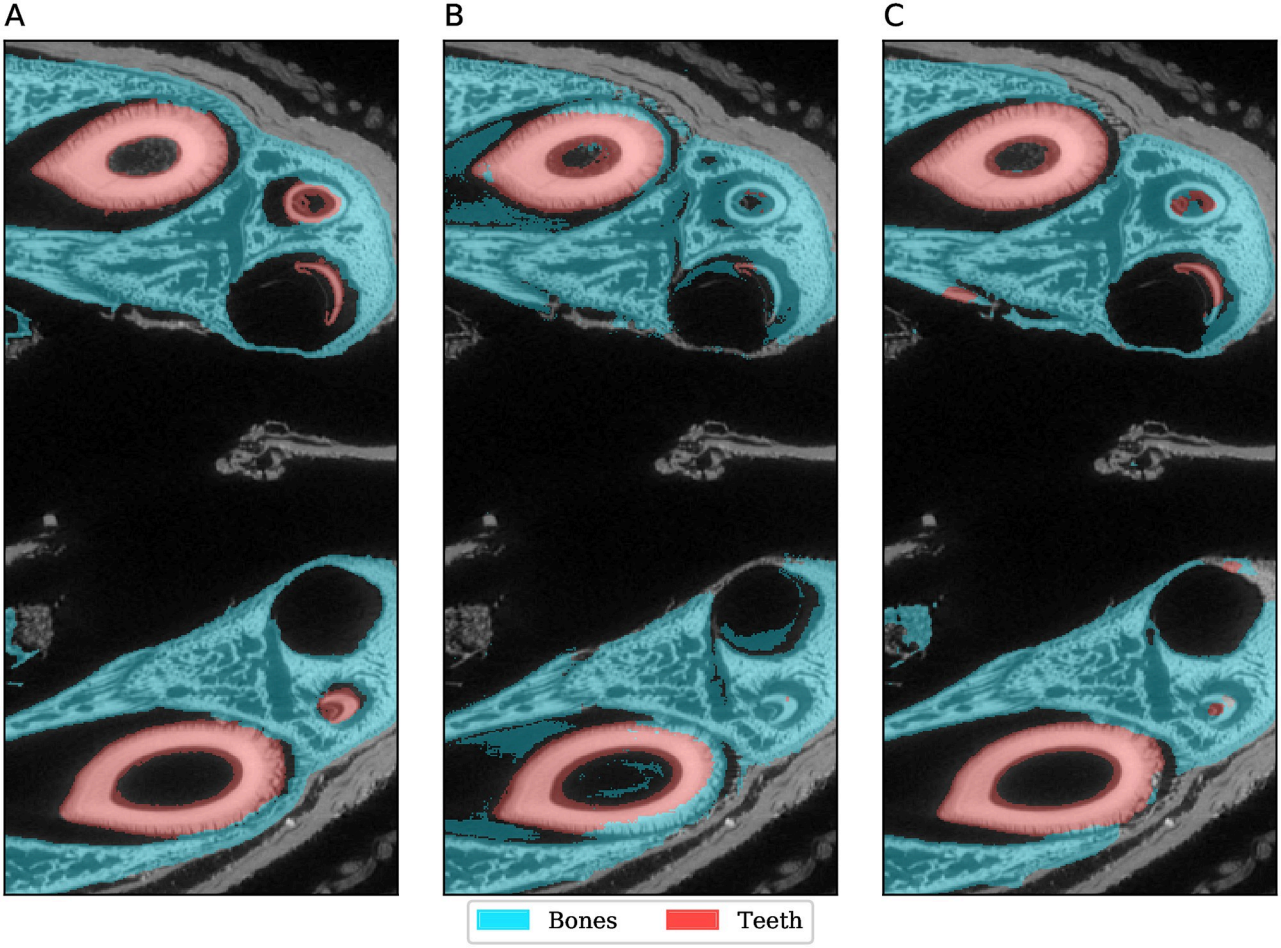
Segmentation detail in MHNGr.ET.1023 (dog). A: manual labelling, B: ASEMI output (random forest classifier), C: U-Net output.

While the numerical accuracy may be lower, a visual inspection of the segmentations shows that the overall output from ASEMI is still useful. In MHNGr.ET.1023 (dog), for example, the largest misclassification is that 26% of the ‘teeth’ voxels were mislabelled as ‘bones’. This is hardly surprising, given the similarity between these materials and the rather low representation of ‘teeth’ in the samples. We also see the inverse error, with ‘bones’ being mislabelled as ‘teeth’, as shown in Fig 7. Where teeth and bones are confused, they are very similar in texture and brightness. The error is not greater than it is only because the bone developed to the same density of teeth in small regions of the skull. Distinguishing between bones and teeth will generally be problematic, due to similarity in the materials’ density and texture. This is an instance where wider context information is likely to be useful. It is interesting that even U-Net made similar errors, indicating that the problem is not a trivial one.

**Fig 8:**
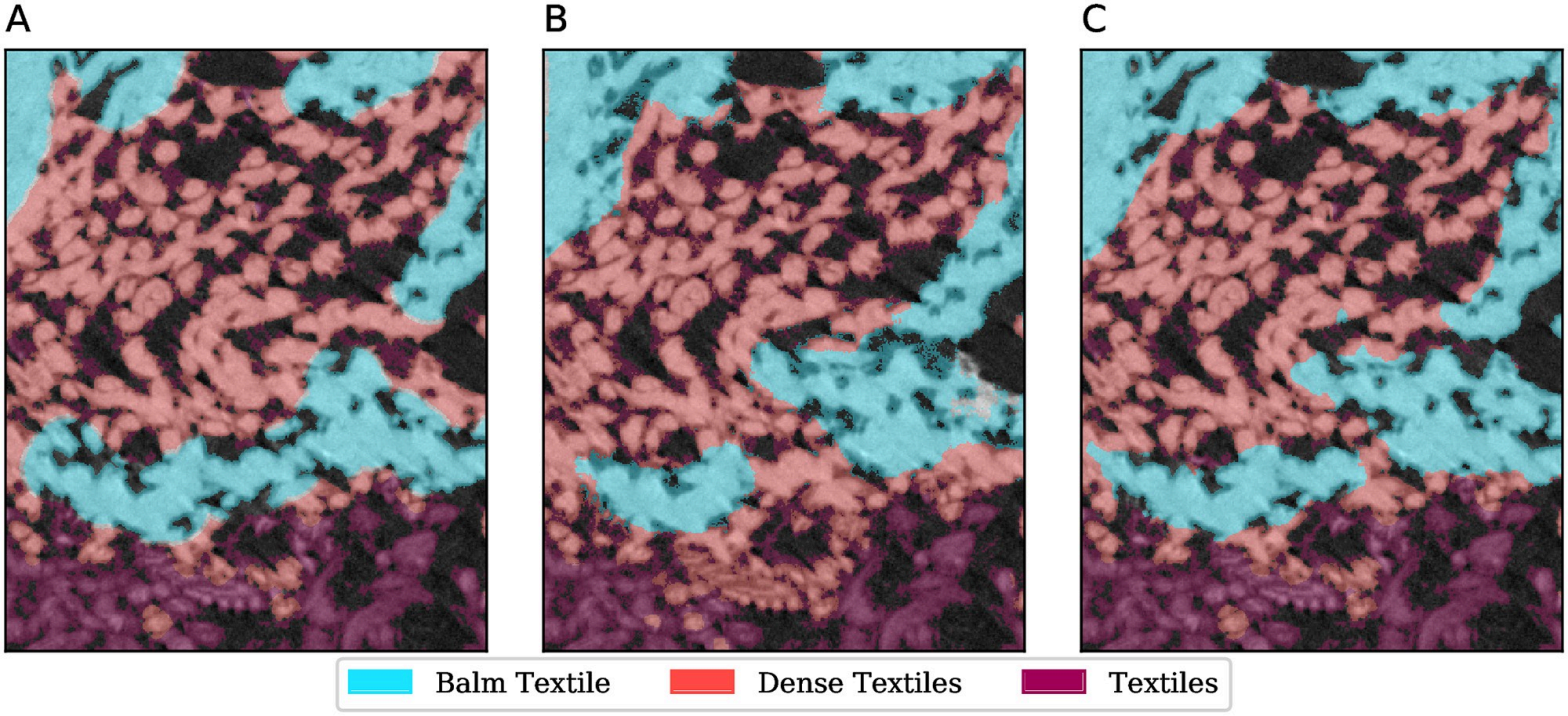
Segmentation detail in MHNGr.ET.1017 (raptor). A: manual labelling, B: ASEMI output (random forest classifier), C: U-Net output.

A similar observation can be made in MHNGr.ET.1017 (raptor), where 14% of ‘wood’ voxels were mislabelled as ‘soft parts’. The ‘wood’ voxels have a low representation, while ‘soft parts’ is a rather diverse collection of voxels, some of which bear similarity to ‘wood’. In this specimen we also observe other examples of mislabelling between similar textures, or where the dividing line is somewhat arbitrary, as shown in Fig 8. In this example we can observe the confusion between ‘textiles’, which is the outer layer of wrapping, ‘dense textile’, which is a denser wrapping, and ‘balm-textile’, which is textile impregnated with embalming resin. In particular, ‘dense textile’ and ‘balm-textile’ have similar density and texture.

**Fig 9:**
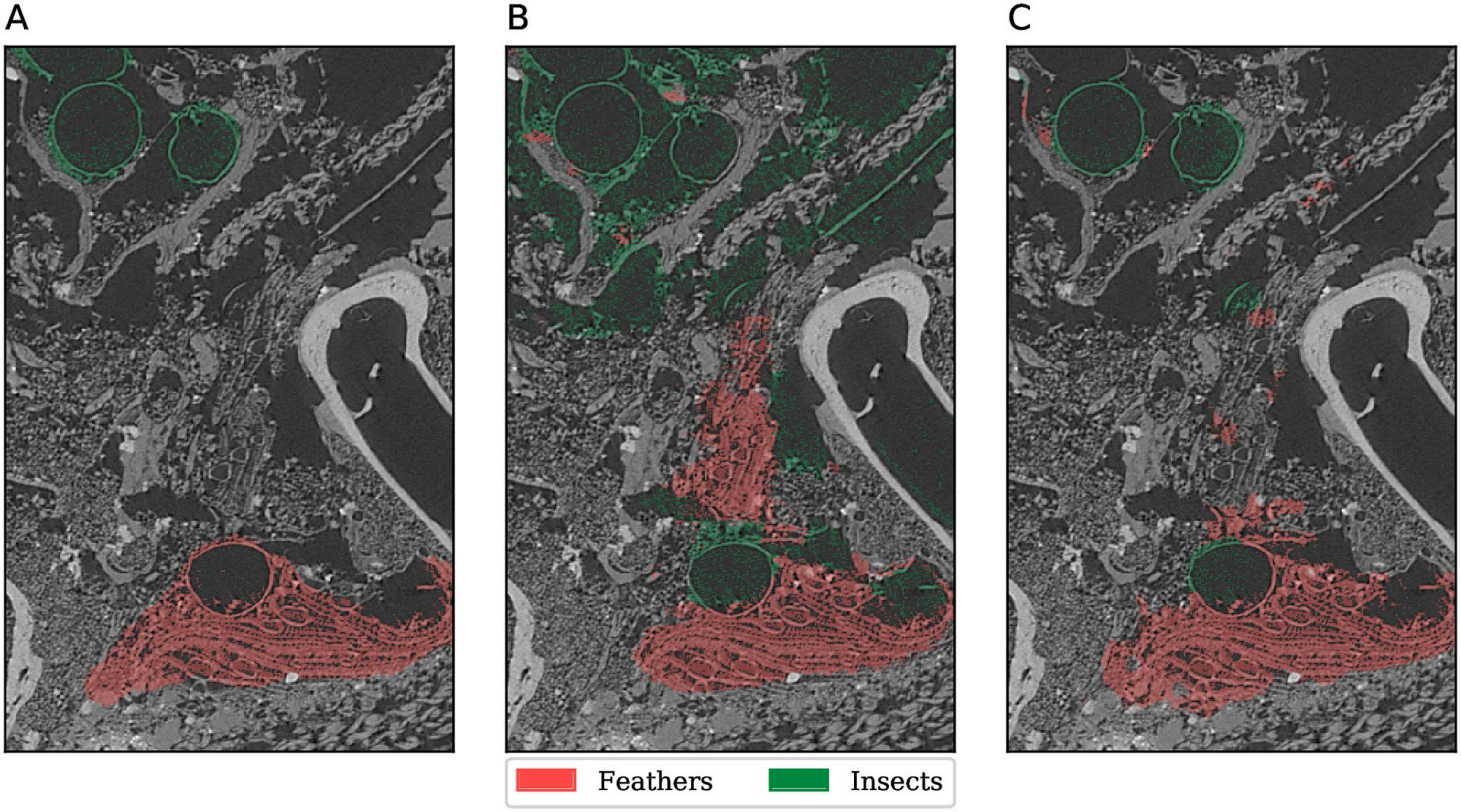
Segmentation detail in MHNGr.ET.1456 (ibis). A: manual labelling, B: ASEMI output (random forest classifier), C: U-Net output.

In MHNGr.ET.1456 (ibis), we see that 12% of ‘natron’, which has a low representation, was misclassified as ‘bones’, which has a similar texture and voxel intensity. Diverse labels, such as ‘soft-powder’, are also easily misclassified to labels that share some texture similarity, such as ‘dense textiles’ or ‘insects’. Another interesting error is the confusion of ‘feathers’ with ‘insects’, as shown in Fig 9. The insects are generally an empty exoskeleton, so that they appear as unfilled circular objects in cross-sections of the volumetric image. Cross-sections of feather stems also appear as unfilled circular objects, perhaps explaining the confusion. As we have seen with similar errors in other specimens, these labels may be distinguished by considering a wider context. Once again, U-Net also made similar errors, indicating the difficulty of the task.

**Fig 10:**
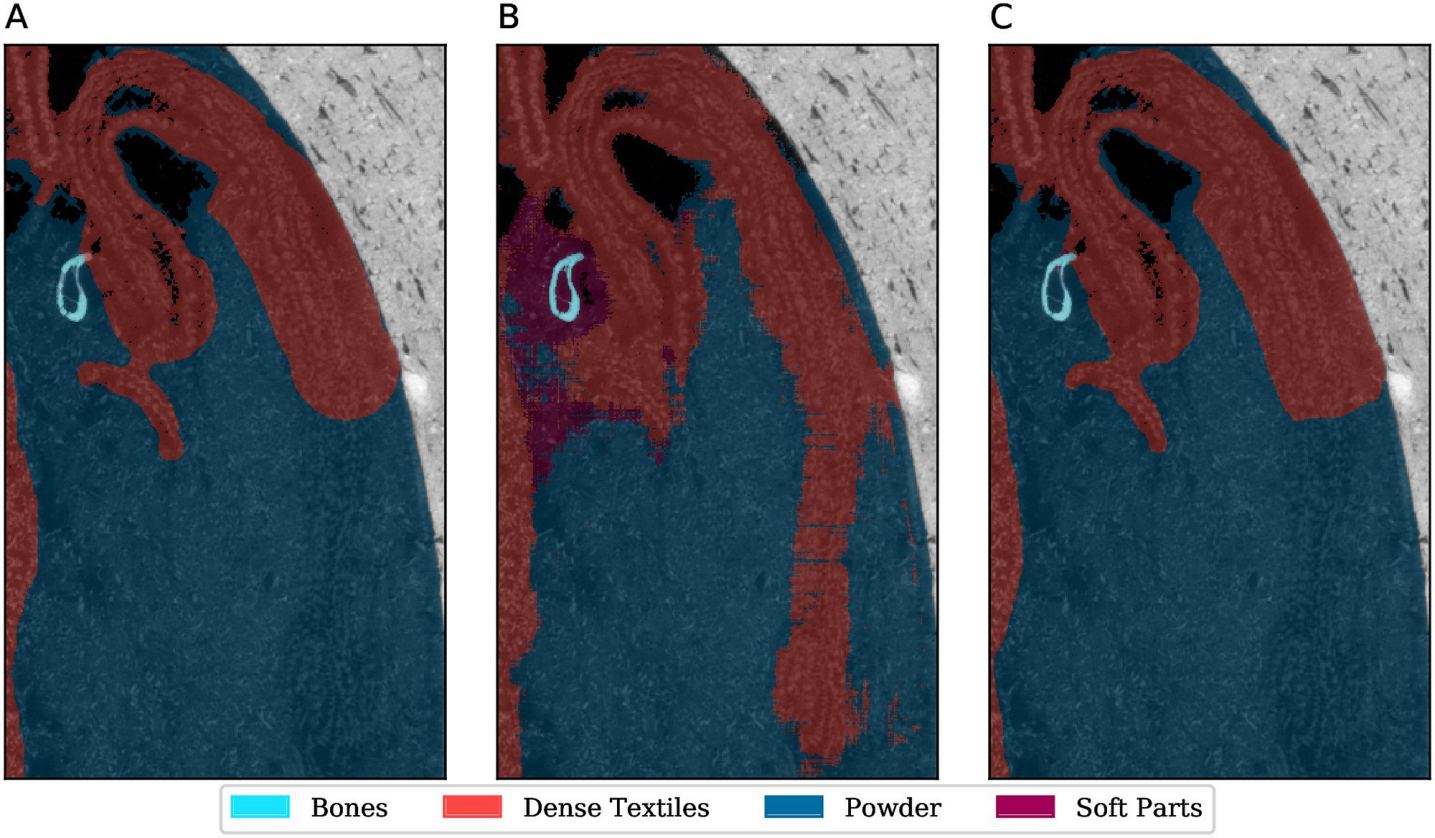
Segmentation detail in MG.2038 (ibis in a jar). Illustrates uncertainty at label edges for the ASEMI segmenter with random forest classifier, some powder being incorrectly labelled as soft parts, and correction of manual mislabelling, as compared to U-Net. A: manual labelling, B: ASEMI output (random forest classifier), C: U-Net output.

Finally, in MG.2038 (ibis in a jar), we see that ‘feathers’, which also have a low representation, misclassify to ‘soft_parts’. There is also misclassification between ‘powder’ and ‘dense textiles’, which have boundaries that are not always cleanly defined in the training set. This is illustrated in Fig 10. It is interesting to see that the reason for this error may be due to a mislabelling in the manual segmentations. The figure shows a section of dense textile which is marked as abruptly ending in the middle of the image, when it seems that it actually extends further down, which the ASEMI prediction corrects. Observe how the U-Net output does not perform this correction, indicating that U-Net is over-fitting to the manual segmentations. A similar smaller error correction occurs in the bone cross-section. Fig 10 also shows evidence of noisy output, particularly in edges between labels. Uncertainty at the edges can be easily fixed using morphological operations; an alternative is to use probabilistic filters, such as Markov random fields.

### 3D rendering of labelled output

**Fig 11:**
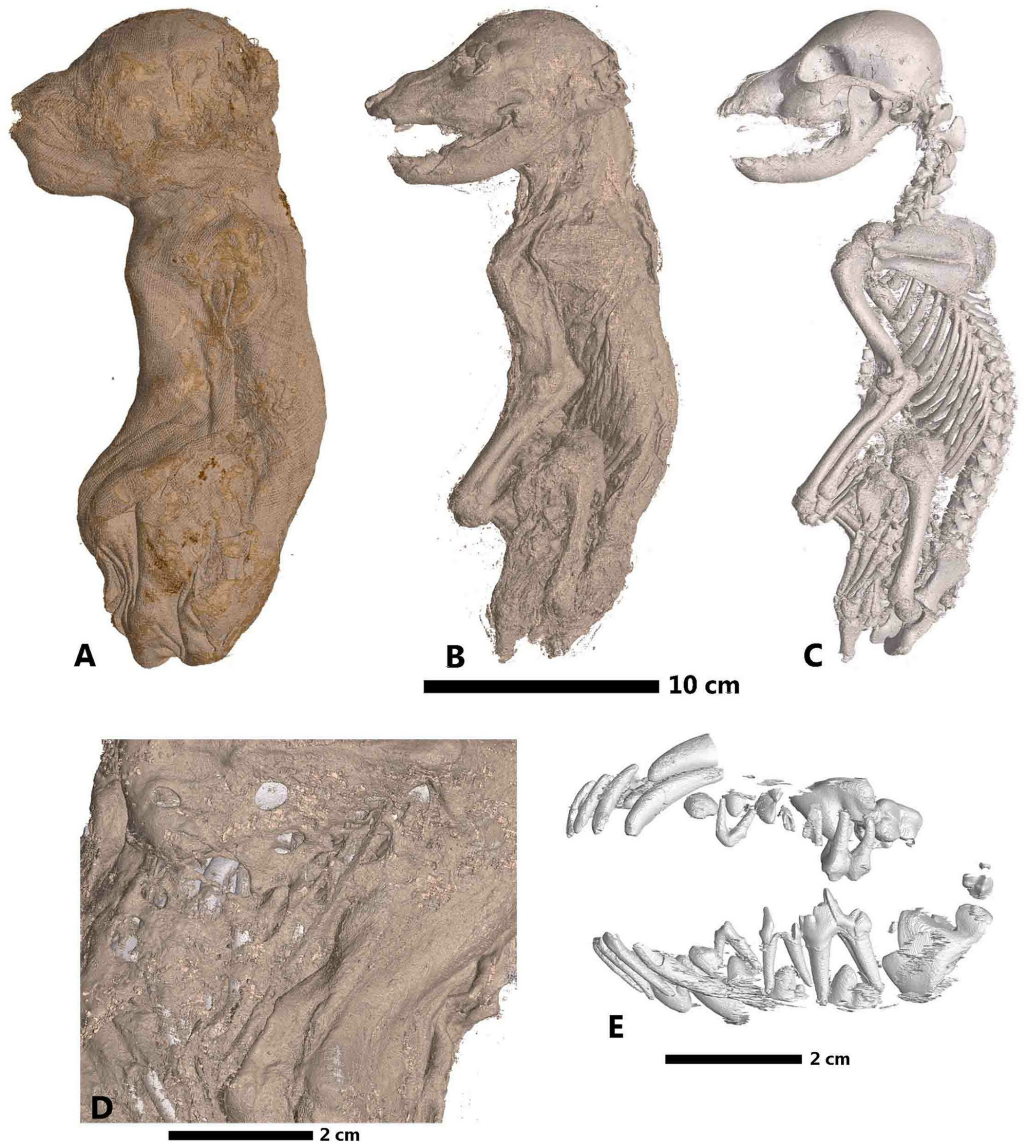
Rendering of the mummy MHNGr.ET.1023 (dog). A: external aspect of the remaining bandages (segmentation ASEMI NN), B: rendering of the preserved soft parts of the puppy (segmentation ASEMI NN), C: rendering of the bones (segmentation U-Net), D: detail on the soft parts showing its weak preservation state through holes certainly caused by pests or putrefaction (segmentation U-Net), E: rendering of the decidual and permanent dentition of puppy showing its young age (segmentation U-Net).

**Fig 12:**
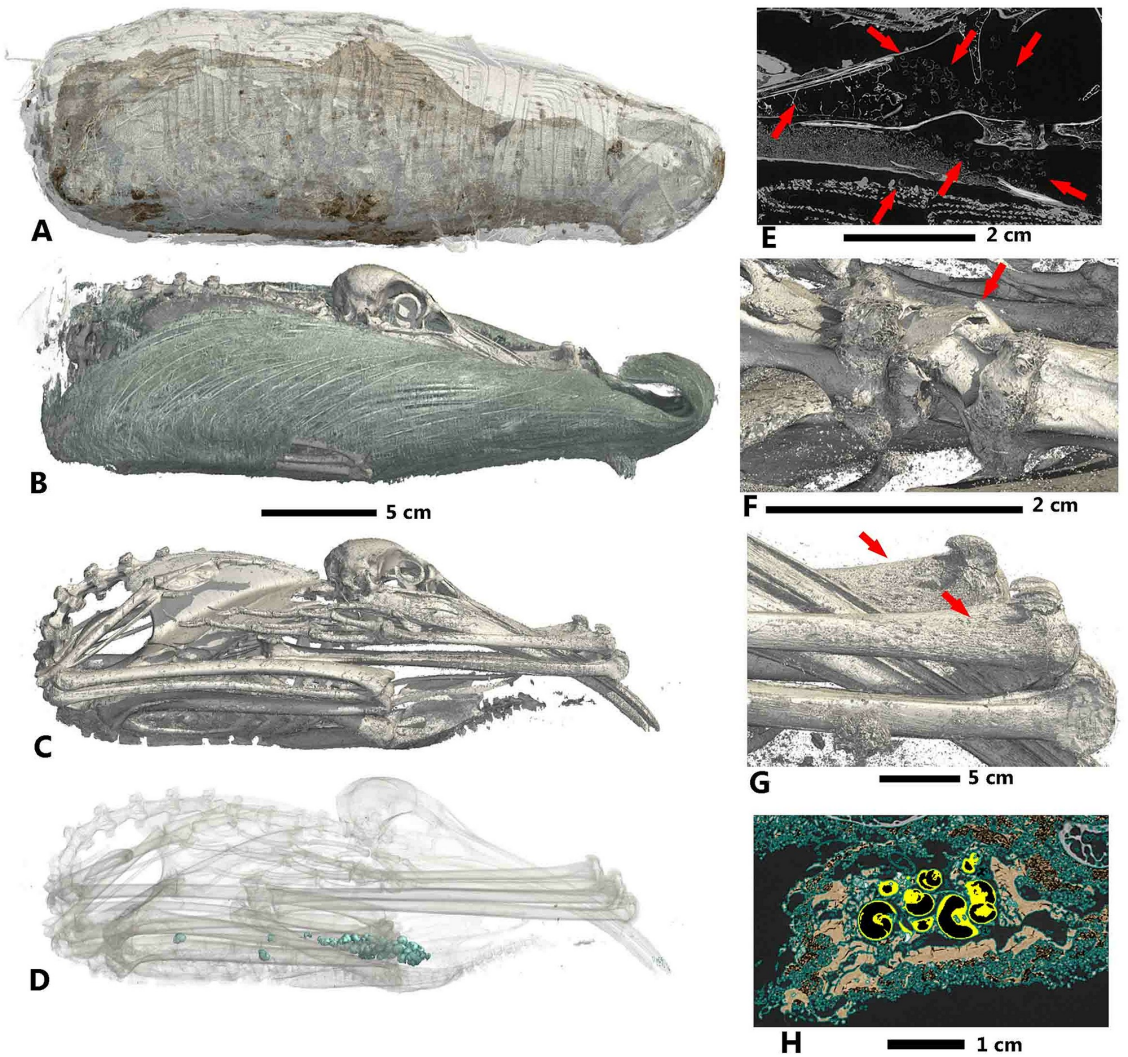
Mummy MHNGr.ET.1456 (ibis). A: general 3D rendering (segmentation ASEMI NN), B: 3D rendering of feathers of the ibis (segmentation U-Net), C: 3D rendering of the skeleton (segmentation U-Net), D: 3D rendering of the gastropods (segmentation U-Net), E: virtual slide showing the pest infestation inside the mummy, F: 3D rendering of the broken vertebra and the twist of the neck (ASEMI NN), G: 3D rendering of the osteoporosis on the forelimbs (ASEMI NN), H: virtual slide of the stomach (orange highlight) and the gastropods (yellow highlight) (segmentation U-Net).

**Fig 13:**
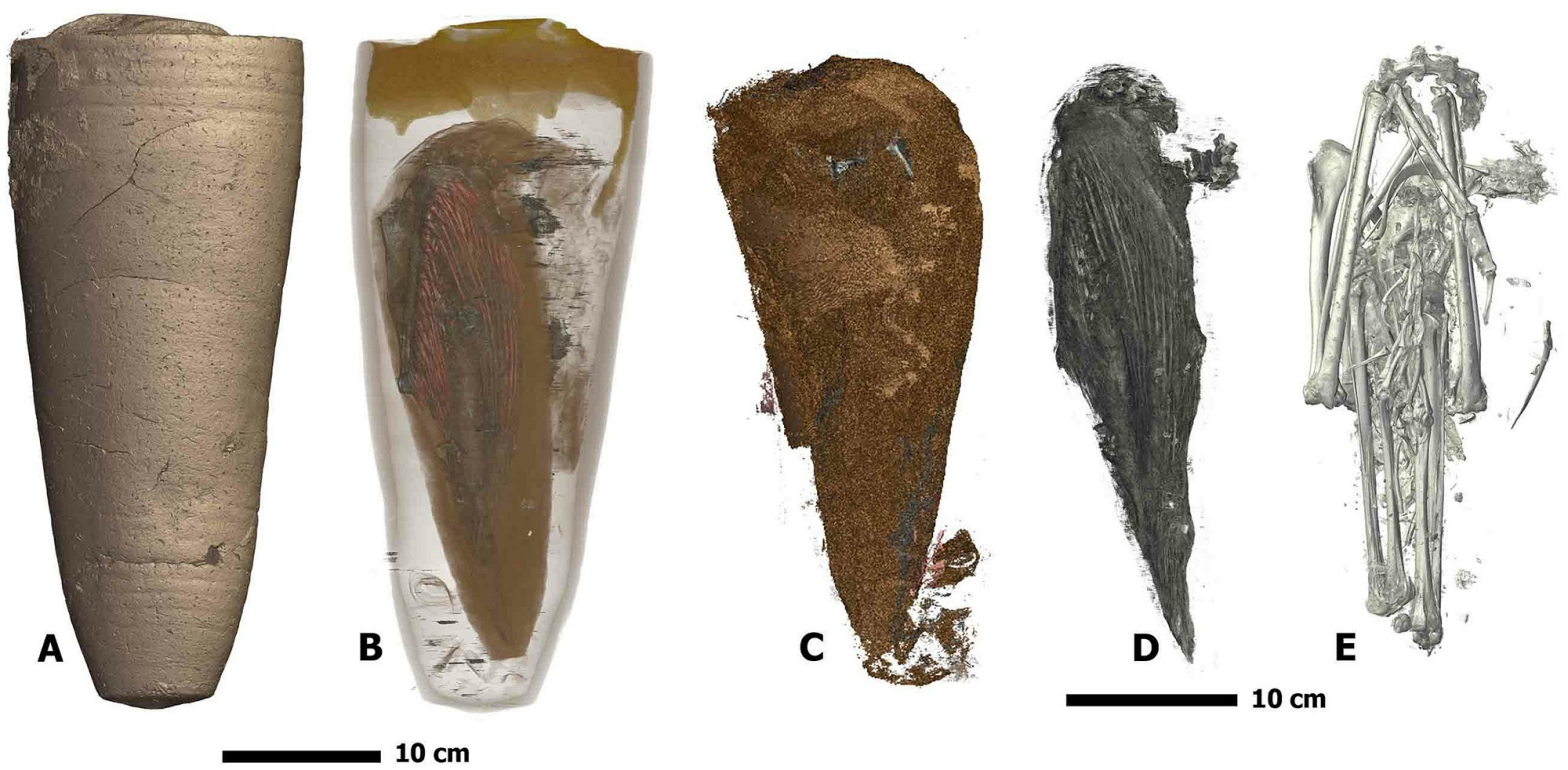
3D rendering of the mummy MG.2038 (ibis in a jar). A: general rendering of the external aspect of the mummy (segmentation ASEMI RF), B: internal content of the mummy made visible thanks to the transparency of the sealed jar (segmentation U-Net), C: focus on the textile surrounding the animal (segmentation ASEMI RF), focus on the ibis inside the mummy D: showing its soft part (segmentation ASEMI NN), E: showing the ibis bones (segmentation ASEMI RF).

Finally, 3D labelled reconstructions of three specimens can be seen in Figs 11–13. For MHNGr.ET.1023 (dog), in Fig 11, the ASEMI segmentation with the neural network classifier is used to render the external wrappings and the soft tissue. Renderings of the bones and teeth use the U-Net segmentation. A detailed image of the soft parts, using the U-Net segmentation, shows the weak preservation state of the mummy, with holes caused by pests or putrefaction. The U-Net segmentation of the teeth (Fig 11E) allows us to estimate its age between six and twelve weeks old. These different segmentations permit us to know that this mummy was made from the decaying corpse of a relatively young puppy. Reconstruction of MHNGr.ET.1456 (ibis) is shown in Fig 12. The ASEMI segmenter with the neural network classifier is used for the general 3D render. The U-Net segmentations was used to image the feathers and the skeleton, as well as the gastropods present inside the stomach of the bird. Using the ASEMI segmenter with the neural network classifier, it was possible to render the neck of the bird, highlighting twisting of the neck and a broken vertebra. The ASEMI segmenter also renders detail of the forelimbs showing osteoporosis. All the segmentations raise questions about the health of the ibis inside the mummy. This can be illustrated by the fact that the cervical fracture may correspond to the cause of the death of the bird. For MG.2038 (ibis in a jar), reconstructions are shown in Fig 13. The ASEMI segmenter with random forest classifier is used for the render of the container. The U-Net segmenter is used to show the interior, by rendering the jar itself transparently. The ASEMI segmenter is used to show the textiles, soft parts, and bones. These different images have brought knowledge on the ibis mummy but also on the jar that contained it. The jar was made on a wheel rotating clockwise and was sealed with a cover glued with a type of mortar. Tomographic data from mummy MHNGr.ET.1017 have shown that the mummy does not contain a complete bird raptor but only its head stuck on the wrapped mummy and fixed through a vegetal stem as well as a very juvenile sea bird in the centre of the fabrics. However, due to the focusing on the internal part of the mummy, the different segmentations show mainly different type of tissues soaked with balm, which make the rendering very confusing for non-specialist eyes.

## Conclusions

In the Automated SEgmentation of Microtomography Imaging (ASEMI) project we have developed a tool to automatically segment volumetric images of Egyptian mummies, obtained using Propagation Phase Contrast Synchrotron Microtomography (PPC-SRμCT). In contrast to emerging commercial solutions, our tool uses simple 3D features and classical machine learning models, for a much lower theoretical complexity. Indicative results were given for four specimens, showing similar overall accuracy when compared with manual segmentations (94–98% as compared with 97–99% for deep learning). A qualitative analysis was also given, showing that our results are close in terms of usability to those from deep learning.

While this work demonstrates the feasibility of using machine learning for the 3D segmentation of large volumes, a number of further advances are necessary for an operational environment. As we have shown, the output segmentations require some postprocessing to smoothen noisy edges. Preliminary work using a Markov Random Field gave promising results, and we plan to implement this in a scalable way. Current 3D deep learning segmentation methods do not scale well to large volumes. If our complexity reduction technique can be applied to established deep learning models, it would become feasible to use deep learning with a full 3D context. This would allow us to get the accuracy we have observed with deep learning models without the discontinuities that exist with current 2D-context approaches. One of the limitations of the current approach is the need for manual segmentation of a small set of slices for training purposes. Extending our segmenter to use an incremental learning approach would allow the iterative improvement of a learned model as further specimens are segmented. This would enable the use of a model trained on one or more specimens to segment a completely new specimen, with reduced user input. Such an extension is necessary for the automatic segmentation of collections of related mummies, which is currently not feasible.
